# Desiccation Risk Drives the Spatial Ecology of an Invasive Anuran (*Rhinella marina*) in the Australian Semi-Desert

**DOI:** 10.1371/journal.pone.0025979

**Published:** 2011-10-17

**Authors:** Reid Tingley, Richard Shine

**Affiliations:** School of Biological Sciences A08, University of Sydney, Sydney, New South Wales, Australia; University of Sao Paulo, Brazil

## Abstract

Some invasive species flourish in places that impose challenges very different from those faced in their native geographic ranges. Cane toads (*Rhinella marina*) are native to tropical and subtropical habitats of South and Central America, but have colonised extremely arid regions over the course of their Australian invasion. We radio-tracked 44 adult cane toads at a semi-arid invasion front to investigate how this invasive anuran has managed to expand its geographic range into arid areas that lie outside of its native climatic niche. As predicted from their low physiological control over rates of evaporative water loss, toads selected diurnal shelter sites that were consistently cooler and damper (and thus, conferred lower water loss rates) than nearby random sites. Desiccation risk also had a profound influence on rates of daily movement. Under wet conditions, toads that were far from water moved further between shelter sites than did conspecifics that remained close to water, presumably in an attempt to reach permanent water sources. However, this relationship was reversed under dry conditions, such that only toads that were close to permanent water bodies made substantial daily movements. Toads that were far from water bodies also travelled along straighter paths than did conspecifics that generally remained close to water. Thus, behavioural flexibility—in particular, an ability to exploit spatial and temporal heterogeneity in the availability of moist conditions—has allowed this invasive anuran to successfully colonize arid habitats in Australia. This finding illustrates that risk assessment protocols need to recognise that under some circumstances an introduced species may be able to thrive in conditions far removed from any that it experiences in its native range.

## Introduction

Human activities have introduced species to areas that lie far outside of their native geographic ranges. Only a small proportion of the global fauna and flora have ever been introduced to a novel environment [1], [2], but many of these translocated species flourish and spread widely in their new ranges [1], [3]–[6]. This is surprising, in that we might expect organisms that have evolved under one set of selective forces (reflecting biotic and abiotic challenges confronted in the native range) to be poorly suited to a different environment that poses a novel suite of challenges [7]. Presumably for this reason, invasion success is highest in ecologically generalized organisms that are introduced to places that experience similar climates to their native ranges [6], [8]–[11]. Yet, some invaders flourish in places that impose challenges very different from those faced in their ancestral ranges [12]. For example, spotted knapweed (*Centaurea maculosa*), an invasive neophyte in North America, inhabits drier conditions in its invaded range than in its native European range [13]. Invasive fire ants (*Solenopsis invicta*) also occupy different climatic niches in their native and invaded ranges [14]. Thus, the absence of ‘suitable’ environmental conditions at a given introduction point may not prevent an invader from thriving at that location once introduced.

**Figure 1:**
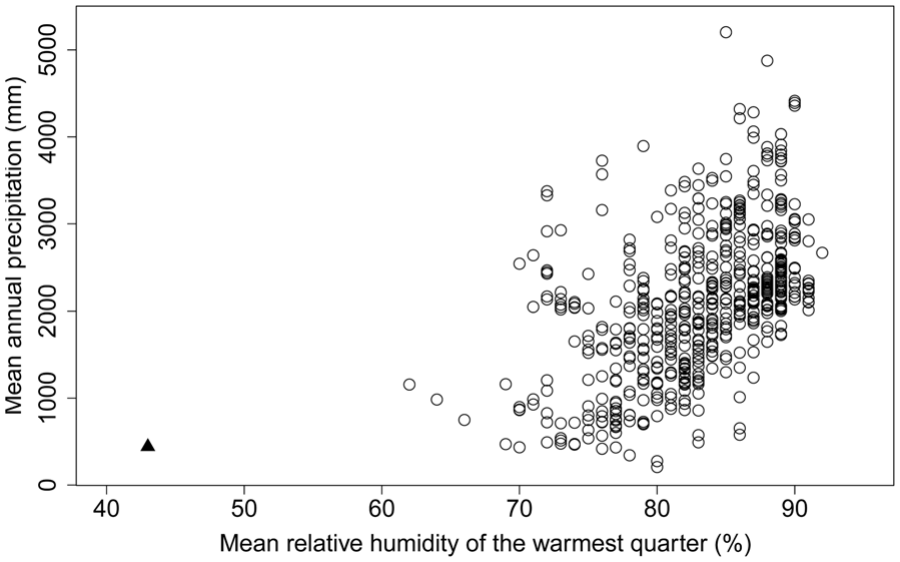
Cane toads in Longreach, QLD (triangle) occupy drier environments than in their native range (circles). Toad occurrence data were taken from museum records (R Tingley & M Kearney, submitted). Climate variables were extracted from the CRU CL 2.0 dataset, which is based on the 1961–1990 normal [39], [40].

One of the most remarkable climatic niche shifts during a biological invasion involves the cane toad, *Rhinella marina*. Cane toads are native to well-watered regions of tropical and subtropical Central and South America. However, this species has not only managed to invade the driest inhabited continent on earth (Australia), but increasingly has penetrated into extremely arid parts of that continent (figure 1), expanding its range to encompass more than 1.2 million km^2^ within seven decades of its introduction in 1935 [15]. Nevertheless, cane toad eggs and larvae are obligately aquatic, and the skin of adult toads acts as a free water surface [16]; thus, water availability is likely the primary factor limiting the range expansion of cane toads in arid systems. Even in the Australian wet tropics (where this constraint is less severe), cane toads select diurnal shelter sites that minimize rates of evaporative water loss, and require frequent access to water to rehydrate [17], [18]. The toad's dependence on open water also endangers many components of the native arid-zone fauna, in that both toads and native species are concentrated around water bodies for extensive periods of the year in arid systems. This high degree of sympatry may present a major threat for native predators in particular, which have no shared evolutionary history with toads, and thus are extremely vulnerable to toad toxins [19]–[21].

Here we present the results of a radio-tracking study conducted at the expanding edge of the cane toad's range in semi-arid Queensland. Our aim was to understand how cane toads deal with the harsh hydric conditions posed by the Australian semi-desert. Specifically, we investigated whether desiccation risk influences patterns of toad shelter site selection and daily rates of movement. Identifying the proximate causes of toad distribution and dispersal in arid regions of Australia may clarify the future impacts of toads through space and time, and may also help focus control efforts to eradicate or slow the spread of toads in arid systems.

## Methods

### Ethics Statement

Permits for this research were provided by the University of Sydney Animal Care and Ethics Committee (permit L04/4-2009/3/4999).

### Study Area

Radio-telemetry was conducted at a cattle station (Whitehill) 30 km south-west of Longreach, Queensland. The first cane toad arrived at Whitehill Station in 2007, although successful reproduction was not observed until 2008. Vegetation within the study area predominately consists of paddocks of Mitchell grass (*Astrebla* spp.). Artificial dams constructed for pastoralism provide the only sources of permanent water throughout the year.

The study area lies on the edge of the Australian arid zone (mean annual rainfall = 447.2 mm, mean annual number of days with rain ≥1 mm = 33.3), and experiences high temperatures year-round (mean monthly maximum temperature = 31.4°C; http://www.bom.gov.au). However, weather conditions during our study were unusually humid (rainfall during tracking period = 326 mm *vs*. 159 mm based on long-term normal). Thus, our study was conducted under conditions that maximized toad dispersal opportunities.

### Radio-telemetry

Between 01-Jan and 03-Mar-2010, 44 adult cane toads (n = 29 males, mean ± S.E. snout-urostyle length = 111 mm±1.27, mass = 167 g±6.98; n = 15 females, 124 mm±3.05, 289 g±27.1) were fitted with single-stage radio-transmitters (Sirtrack Ltd., Havelock North, NZ). Radio-transmitters were attached to a flexible metal chain around each toad's waist, and did not impede movement or amplexus (transmitter mass ≤3.5% of toad body mass). Toads were released immediately after capture, and located every 1 to 3 days (median = 1 day) for a total of 2 to16 days (median = 7 days). Toad locations were recorded with a handheld GPS unit (*c*. 5 m accuracy, Garmin Ltd., Oregon, USA). We calculated daily movement rates by dividing the straight-line distance between successive toad locations by the number of days that had elapsed since last capture. Adult cane toads move about only at night, so our calculations of daily movement rates are based only on diurnal shelter site locations [22].

### What Factors Drive Cane Toad Dispersal?

We investigated whether three abiotic variables influenced daily movement rates of radio-tracked toads after accounting for snout-urostlye length (SUL): (i) mean nightly temperature, (ii) number of days since rain, and (iii) distance to the nearest water body. These three variables were selected based on personal observations, and the results of previous studies [23]–[25]. Mean nightly temperature data (averaged from sunset to sunrise, using data on times from: http://www.usno.navy.mil/USNO/astronomical-applications/data-services/rs-one-year-world) were collected with hygrochon data loggers (Dallas Semiconductor, Texas, USA) placed around the perimeter of each water body. Rainfall data were gathered with a rain gauge located in the middle of the study site.

To determine whether abiotic variables affected (log) movement rates of toads, we used linear mixed effects models and an information-theoretic approach. Akaike's information criterion (AIC) and Bayesian information criterion (BIC) were used to rank the goodness of fit of all possible models containing SUL and the three abiotic variables. These two measures of model parsimony provide upper and lower bounds on model complexity [26]: use of AIC typically results in overly complex models, whereas BIC often selects models that underfit the data (because the penalty for additional parameters is stronger in BIC than in AIC). To facilitate interpretation and reduce the chance of over-fitting models, only two-way interactions between variables were considered. A random effect was also included in all models to account for clustering of movement rates within individual toads; SUL and all abiotic variables were treated as fixed effects. Squared Pearson correlations between observed and predicted movement rates were used to measure the explanatory power of candidate models.

We also investigated whether the straightness of toad movement paths was influenced by the mean distance to the nearest water body. Path straightness was calculated by dividing the net displacement between each toad's initial and final location by the sum of daily movement distances [22]. Perfectly straight paths have a value of 1, whereas paths that start and end at the same location have a value of 0. Only toads that were captured at least four times (n = 33) were included in these analyses. For each toad, we calculated the mean distance to the nearest water body across all locations. We then used a t-test to compare the straightness of movement paths between toads that were close to (<18.6 m), or far from (≥18.6 m) water bodies, based on the median distance to water bodies across all toads.

### What Factors Drive Cane Toad Shelter Site Selection?

We used plaster casts of an adult cane toad in the water-conserving posture to quantify desiccation rates in diurnal shelter sites. To mimic the total absorptivity of a cane toad, powder paint was mixed with the plaster before allowing each model to set [27]. A layer of flexible rubber (Performix Plasti Dip, New South Wales, AU) was applied to the undersides of all models to prevent water exchange between models and the substrate. These plaster models lose water at a rate similar to that of live cane toads under field conditions [27]. Models were submerged in water for 24 hours, weighed with a spring scale, and subsequently placed in pairs of shelter sites (used and randomly selected) for *c*. 24 hours before being reweighed. Random locations were selected by spinning the dial of a compass and randomly choosing a distance between 1 and 10 m from each used shelter site. Canopy cover and the maximum height of the closest vegetation were also measured to investigate whether differences in rates of evaporative water loss between sites could be attributed to these characteristics. Canopy cover was measured by placing a plaster model of a toad in each retreat site. Looking from above, we visually estimated the percent of the model that was covered by vegetation.

Differences in rates of evaporative water loss from plaster models placed in used *vs*. random shelter sites were analysed with a paired t-test. We used linear regressions to investigate whether canopy cover and vegetation height influenced rates of evaporative water loss. All statistical analyses were conducted in R^©^ 2.12.0 [28].

## Results

**Figure 2:**
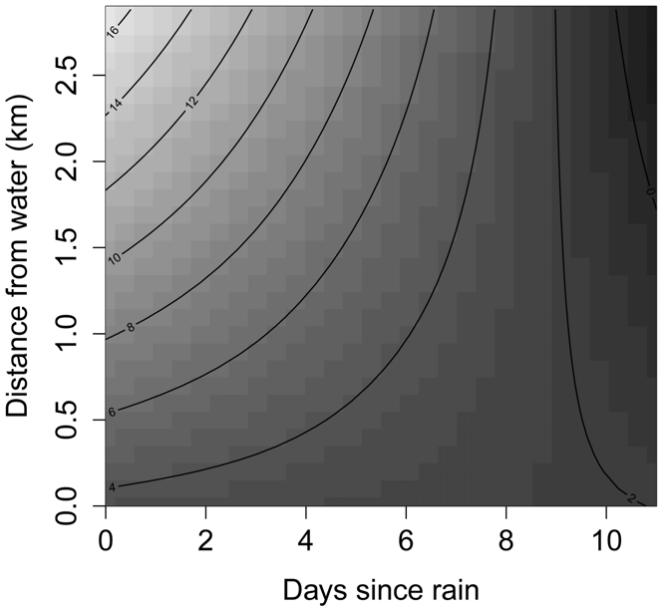
Hydric constraints on cane toad movement rates. Shown is the relationship between log-movement rate, the number of days since rain, and the distance to the closest water body. The interaction between these variables was estimated using a tensor product smooth term within a generalized additive mixed model (estimated degrees of freedom = 3.01). Contours and shading represent different values of the response variable (log-movement rate, m day^−1^).

Toad movement rate decreased as the number of days since rain increased (i.e., decreased with increasing aridity), but this correlation was modified by an interaction with the distance to the closest water body. Under wet conditions, toads that were far from water moved further than did conspecifics that remained close to water, but this relationship was reversed under dry conditions (figure 2). This interaction between rainfall and distance to water explained 24.1% of the variation in toad movement rate.

**Figure 3:**
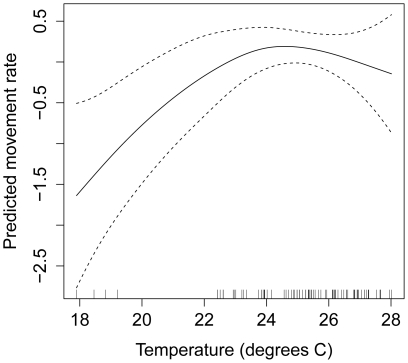
Predictions of cane toad movement rate as a function of mean nightly temperature. This relationship was estimated using a generalized additive mixed model (estimated degrees of freedom = 1.79). Predictions are shown on the scale of the linear predictor, with larger values indicating farther movements.

We also found support for a quadratic relationship between daily movement rates of toads and mean nightly temperature. Movement rate increased with increasing temperature until *c*. 25°C, but began to slightly decrease above this threshold (figure 3). Inclusion of a quadratic temperature term explained an additional 4.40% of the variance in toad movement rate (pseudo-R^2^ = 28.5%). A model containing a quadratic term for temperature and an interaction between the number of days since rain and distance to water also had reasonably high support according to AIC and BIC (table 1). These findings were robust to the inclusion of snout-urostyle length, which had a weak (pseudo-R^2^ = 28.6%; table 1) negative effect on daily movement rates (i.e., larger toads moved shorter distances).

**Table 1:** Highest-ranked models of cane toad movement rate.

Rain	Dwater	Rain*Dwater	Temp^2^	SUL	ΔAIC	wAIC	ΔBIC	wBIC
−0.139	0.00460	−0.000521	3.29–4.74		0	0.666	0	0.525
−0.131	0.00464	−0.000527	3.68–4.64	−0.00828	1.56	0.306	4.77	0.0480
−0.138	0.00483	−0.000546			6.99	0.0200	0.556	0.397

Shown are unstandardized parameter estimates, the difference in Akaike (AIC) and Bayesian information criterion (BIC) between each model and the highest ranked model (ΔAIC and ΔBIC, respectively), and the Akaike (wAIC) and Bayesian weights (wBIC) of each model. Rain = number of days since rain, Dwater = distance to closest water body, Temp^2^ = quadratic effect of mean nightly temperature, SUL = snout-urostyle length.

Desiccation risk also influenced the tortuosity of toad movement paths. Toads that were far from water bodies travelled along straighter paths (mean straightness ± S.E. = 0.641±0.0751) than did conspecifics that generally remained close to water (mean straightness ± S.E. = 0.415±0.0778; *t*_30.9_ = 2.09, *P* = 0.0449).

**Figure 4:**
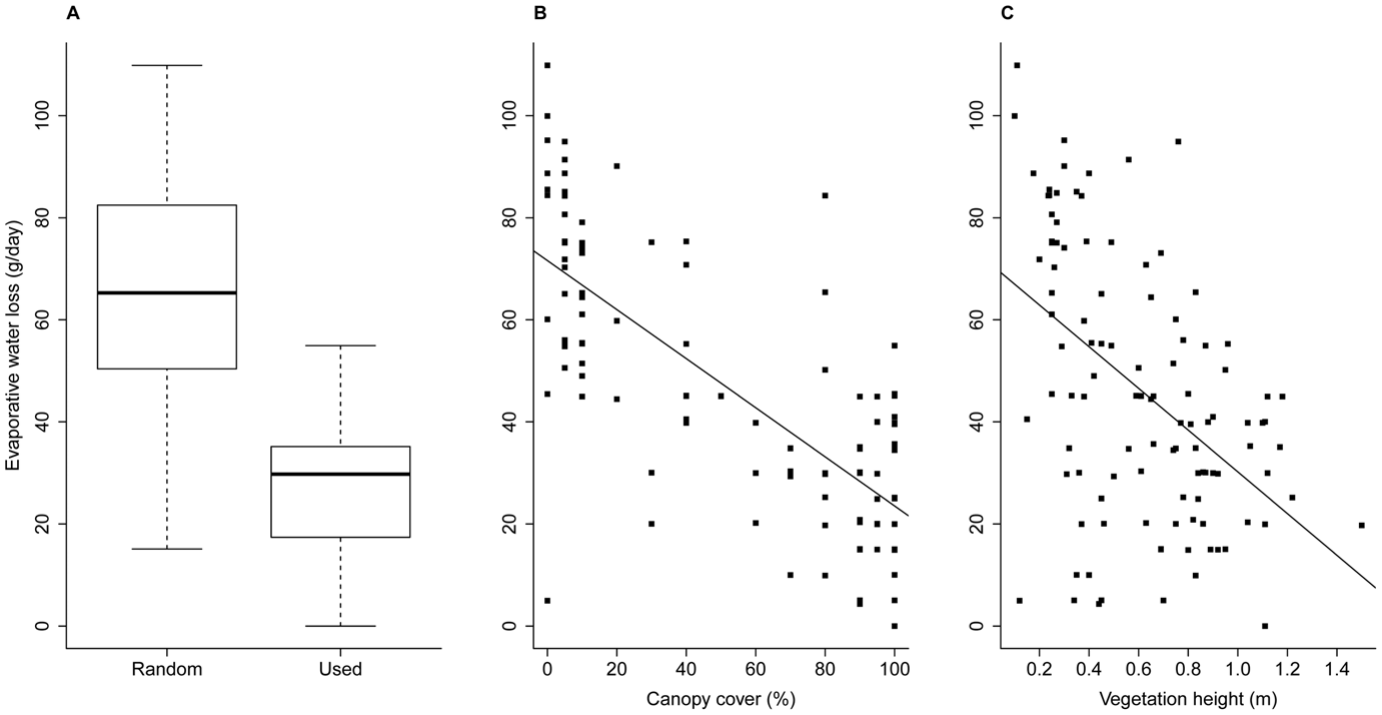
Characteristics of cane toad shelter sites. Plaster models of adult cane toads placed in diurnal shelter sites had lower rates of evaporative water loss than did models placed in random locations (a). These differences between sites were due to (b) variation in canopy cover and (c) vegetation height.

Patterns of toad shelter site selection were driven by desiccation risk as well; plaster models placed in diurnal shelter sites had lower rates of evaporative water loss than did models placed in random locations (mean difference = 38.6 g day^−1^, *t*_54.0_ = 13.6, *P*<0.0001, figure 4A). Toads sheltered in shallow depressions, cracks, or burrows 18.2% of the time, but these subterranean shelters did not provide lower desiccation rates than shelter sites on the surface (evaporative water loss rates in toad shelters above *vs*. below ground: *t*_11.4_ = −2.02, *P* = 0.0678). Importantly, differences in rates of evaporative water loss between used and randomly selected shelter sites were not due to the fact that toads sometimes sheltered below ground (mean difference after exclusion of subterranean shelters = 33.6 g day^−1^, *t*_44.0_ = 12.8, *P*<0.0001). Instead, differences in rates of evaporative water loss between used and random sites were due to variation in canopy cover and vegetation height. Rates of evaporative water loss decreased with increasing canopy cover (*F*_1, 108_ = 130, *P*<0.0001, R^2^-adjusted = 0.542, figure 4B) and vegetation height (*F*_1, 108_ = 32.9, *P*<0.0001, R^2^-adjusted = 0.226, figure 4C).

## Discussion

Risk assessment schemes are widely used to predict which alien species should be targeted for quarantine and eradication [29], [30]. However, most risk assessments treat alien species as if they are fixed entities, ignoring the fact that microevolution or phenotypic plasticity may allow aliens to invade environments that lie far outside of their ancestral niches [12], [31]. In animals, behavioural plasticity has been shown to be particularly important in determining invasion success. For example, introduced vertebrates with larger relative brain sizes (and thus greater behavioural flexibility) have been more successful at establishing viable populations than have smaller-brained species [32]–[34]. Our results go one step further and demonstrate that behavioural plasticity can also modify the rate of spread and eventual geographic distribution of an invader. In particular, patterns of cane toad movement and shelter site selection suggest that an ability to exploit spatial and temporal heterogeneity in the availability of surface water has facilitated the range expansion of this anuran invader in the Australian semi-desert.

Unlike some highly specialised arid-zone lineages of anurans, toads (like many forest-origin amphibians) have highly permeable skins, and lack morphological or physiological means to reduce rates of evaporative water loss [19], [24]. Our results demonstrate that cane toads in semi-arid systems compensate for this maladaptation by retreating to damp shelter sites by day, and sometimes remaining in these diurnal shelters for days or weeks at a time during prolonged periods of drought. In addition, toads in semi-arid landscapes further minimize desiccation risk by moving long distances between shelter sites only when hydric conditions permit. After it rains, toads which are far from water take advantage of favourable conditions by rapidly moving between shelter sites, presumably in an attempt to reach permanent water sources. However, as the landscape dries out, toads that remain far from water cease moving about, and only toads that are close to permanent water bodies continue to make substantial daily movements. Semi-arid cane toads also reduce the amount of time that they are exposed to desiccating conditions by moving along relatively straight paths when they are far from water bodies. Simulations have shown that such linear dispersal is a highly efficient search strategy for animals attempting to locate suitable habitats in unfamiliar terrain [35]. This suite of water-conserving tactics will be particularly important for toads under conditions of more typical (low) rainfall. In most years, effects of desiccation risk on shelter site selection and movement thus will be stronger than were observed during this study.

Our finding that desiccation risk drives patterns of shelter site selection in cane toads is consistent with previous studies conducted in tropical Queensland [17], [18]. Even in these more mesic environments, toads actively selected shelter sites that reduced rates of evaporative water loss. However, our results concerning effects of desiccation risk on daily movement rates differ from those revealed by studies in the Australian tropics. In the wet-dry tropics of the Northern Territory, daily movement rates of toads during the wet season were significantly correlated with abiotic variables [25], but these variables had low explanatory power (R^2^ of highest ranked model = 5%). Similarly, in tropical Queensland, a previous radio-tracking study found no significant relationship between abiotic variables and daily movement parameters of cane toads [24]. Thus, desiccation risk appears to have a more marked influence on the movements of cane toads in semi-arid landscapes than in tropical regions. Nonetheless, over 70% of the variation in toad movement rate in our study was unexplained by thermal and hydric constraints. This unexplained variation has several potential sources. For example, radio-telemetry likely underestimated the distances that toads moved between relocations, obscuring relationships between abiotic variables and movement rates. Additionally, expanding the period of data collection to include cooler and drier periods of the year would improve the explanatory power of our models. Future research also could usefully explore the evolutionary underpinnings of the observed movements (e.g., by comparing movement parameters between populations that differ in time since colonization [22]).

Our analyses also revealed that toad movement rates were influenced by mean nightly temperature, although this relationship was much weaker than that between movement rates and the interactive effects of rainfall and distance to water. This finding is consistent with biophysical analyses of the cane toad's fundamental niche, which suggest that activity and movement of toads throughout much of arid Australia are not severely constrained by temperature [36]. In contrast, ambient temperatures determine rates of toad activity and spread in southern areas of the toad's Australian range [31], [36].

The results of the current study are of interest not only in understanding how behavioural flexibility allows species to invade novel environments, but also for predicting spatial and temporal variation in the impact of cane toads on native arid-zone fauna. Our finding that desiccation risk constrains the spatial ecology of toads suggests that native species that congregate around water bodies may be especially at risk in arid systems. The likelihood of encounters between toads and native species will be particularly high during droughts, when both toads and native species are forced to remain near water bodies for extensive periods. However, during the wet season, our results illustrate that toads have the ability to spread widely throughout the landscape (>2.8 km from water), and thus encounter (and potentially kill) a wider suite of predators (e.g., goannas, snakes) and prey (e.g., invertebrates) [37]. The effects of cane toads on the native fauna of the arid zone therefore will vary through both space and time, with a high probability of impact around water bodies during periods of drought, and a broader impact across the landscape following rainfall.

Finally, our results also have management implications with respect to the rate of spread and eventual geographic range of cane toads in arid Australia. Toad movement and distribution are largely dictated by the availability of free water, and this dependence will dramatically curtail the toads' ability to invade deeper into the arid zone. However, the provision of abundant artificial water sources for pastoralism will partially overcome this constraint. Decreasing the connectivity of artificial water bodies therefore may offer the most viable management strategy to limit the distribution of toads and slow their rate of spread into arid systems [38].

The cane toad's conquest of semi-arid Australia represents a remarkable example of how invasive species can colonize environments that lie outside of their native climatic niches. Although the degree of climate-match between a species' native and invasive ranges remains a critical predictor of invasion success [6], [8]–[11], this anuran invader is able to flexibly adapt to harsh abiotic conditions by facultatively modifying its behaviour, in order to exploit spatial and temporal heterogeneity in the availability of suitable habitats. Such behavioural plasticity offers a cautionary tale: risk assessment protocols need to recognise that under some circumstances, an introduced species may be able to thrive in conditions far removed from any that it experiences in its native range.
